# Coverage and determinants of influenza vaccine among pregnant women: a cross-sectional study

**DOI:** 10.1186/s12889-019-7172-8

**Published:** 2019-07-05

**Authors:** Vittoria Offeddu, Clarence C. Tam, Tze Tein Yong, Lay Kok Tan, Koh Cheng Thoon, Nicole Lee, Thiam Chye Tan, George S. H. Yeo, Chee Fu Yung

**Affiliations:** 1 Saw Swee Hock School of Public Health, National University of Singapore and National University Health System, Singapore, 117549 Singapore; 2 London School of Hygiene & Tropical Medicine, London, WC1E 7HT UK; 3 Singapore General Hospital, Singapore, 169608 Singapore; 4 KK Women’s and Children’s Hospital, Singapore, 229899 Singapore; 5 Lee Kong Chian School of Medicine, NTU Imperial College, Singapore, 636921 Singapore

**Keywords:** Influenza, Influenza vaccine, Maternal vaccination, Pregnancy, Vaccine recommendation

## Abstract

**Background:**

Pregnant women are at increased risk of influenza-related complications. The World Health Organisation recommends influenza vaccination to this high-risk population as highest priority. However, achieving high influenza vaccine coverage among pregnant women remains challenging. We conducted a cross-sectional survey to estimate the coverage and determinants of influenza vaccination among pregnant women in Singapore.

**Methods:**

Between September and November 2017, pregnant women aged ≥21 years were recruited at two public hospitals in Singapore. Participants completed an anonymous, self-administered online questionnaire assessing participants’ influenza vaccination uptake, knowledge of and attitudes towards influenza and the influenza vaccine, vaccination history, willingness to pay for the influenza vaccine, and external cues to vaccination. We estimated vaccine coverage and used multivariable Poisson models to identify factors associated with vaccine uptake.

**Results:**

Response rate was 61% (500/814). Only 49 women (9.8, 95% Confidence Interval (CI): 7.3–12.7%) reported receiving the vaccine during their current pregnancy. A few misconceptions were identified among participants, such as the belief that influenza can be treated with antibiotics. The most frequent reason for not being vaccinated was lack of recommendation. Women who were personally advised to get vaccinated against influenza during pregnancy were 7 times more likely to be vaccinated (prevalence ratio (PR) = 7.11; 95% CI: 3.92–12.90). However, only 12% of women were personally advised to get vaccinated. Other factors associated with vaccine uptake were vaccination during a previous pregnancy (PR = 2.51; 95% CI: 1.54–4.11), having insurance to cover the cost of the vaccine (PR = 2.32; 95% CI: 1.43–3.76), and higher vaccine confidence (PR = 1.62; 95% CI: 1.30–2.01).

**Conclusions:**

Influenza vaccination uptake among pregnant women in Singapore is low. There is considerable scope for improving vaccination coverage in this high-risk population through vaccination recommendations from healthcare professionals, and public communication targeting common misconceptions about influenza and influenza vaccines.

**Electronic supplementary material:**

The online version of this article (10.1186/s12889-019-7172-8) contains supplementary material, which is available to authorized users.

## Background

Pregnant women [1, 2] and new-borns [3–5] are at increased risk of complications from influenza infection. Immunisation with seasonal influenza vaccine during pregnancy is associated with lower incidence of influenza infection among pregnant women [6–10] and their new-borns up to six months of age [6–11].

In Singapore, influenza transmission occurs year-round, with two peaks of increased activity coinciding with the northern and southern hemisphere influenza seasons [12]. Since 2014, Singaporean residents in influenza high-risk groups can claim for the influenza vaccine using Medisave, a mandatory medical savings scheme [13]. Pregnant women of all gestational stages are recommended to get vaccinated against influenza [14, 15]. However, currently no data are available on influenza vaccine coverage among pregnant women. Coverage among other influenza high-risk groups, such as elderly [16] and children under five years [17], is low (< 15%).

This cross-sectional survey aimed to i) determine the level of influenza vaccine coverage among pregnant women in Singapore, ii) investigate pregnant women’s knowledge, attitudes and practices towards influenza vaccination in the local context, and iii) identify factors associated with influenza vaccine uptake in this population. Our secondary objective was to compare uptake levels of influenza vaccine with coverage levels of other vaccines in the same population, including the tetanus, diphtheria, and pertussis vaccine, which is also recommended to pregnant women in Singapore, as well as the Hepatitis A and B and meningococcal vaccines.

## Methods

### Recruitment

Between September and November 2017, we recruited pregnant women attending Obstetrics and Gynaecology (OBGYN) clinics at KK Women’s and Children Hospital (KKH) and Singapore General Hospital (SGH) in Singapore. These two hospitals provide care for over a third of pregnancies in Singapore, and offer both public subsidised and private healthcare options. Trained data collectors were allocated to selected antenatal clinics at both hospitals. Apart from the inclusion of both private subsidised and private clinics, no other specific criteria were used for the clinic selection. Four data collectors visited the clinics at KKH on the two week days with the highest expected number of pre-natal appointments. On the remaining week days, recruitment was conducted at OBGYN clinics at SGH. Recruitment was suspended on days when other, unrelated activities were planned at the clinics. Data collectors approached all patients registering for an appointment in the clinic waiting room. Non-pregnant women and women younger than 21 years were excluded. Eligible individuals were asked to provide their informed consent through an online survey form. Consenting participants filled out an anonymous, self-administered online questionnaire on an electronic tablet. Based on previous literature, we expected vaccination prevalence estimates in the range of 10 to 70%. We estimated that a sample size of 500 participants was required to be able to estimate vaccine coverage values in this range with margins of error between ±2.5 and ± 4.5% at a confidence level of 95%.

### Questionnaire

**Fig. 1:**
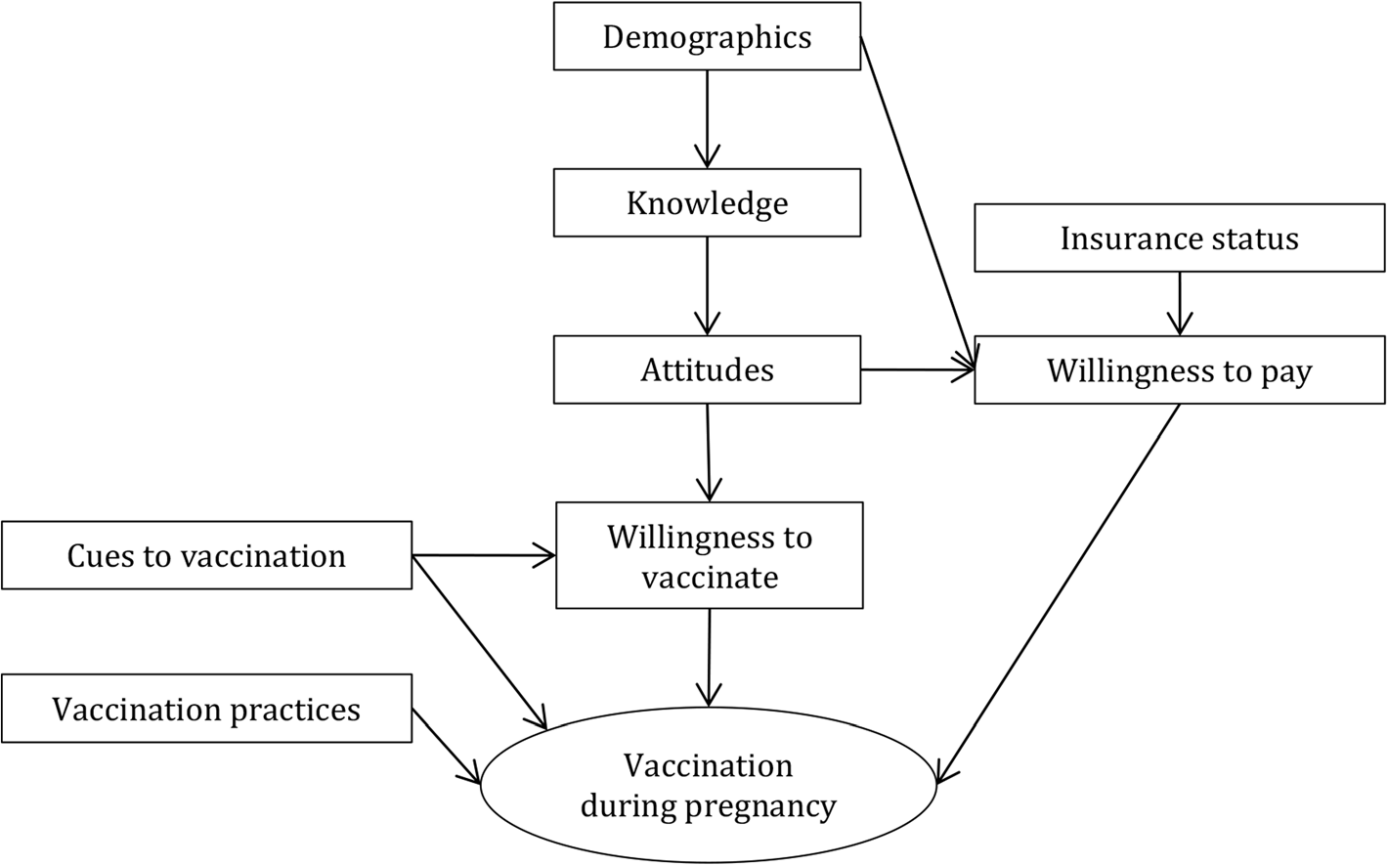
Conceptual framework. Factors contributing to participants being vaccinated against influenza during current pregnancy; for variables under each domain, see Additional file 1 Tables S4 and S5

Our conceptual framework was based on the Health Belief Model of health behaviour (Fig. 1) [18]. The questionnaire included domains assessing participants’ demographics, vaccination history, knowledge, attitudes, insurance status, willingness to pay, cues to vaccination, and willingness to vaccinate (Additional file 1: Table S1).

Participants’ knowledge level was assessed through 3 multiple-choice questions on influenza and 3 single-choice questions on the influenza vaccine (Additional file 1: Table S2). Total knowledge score was calculated as the sum of individual question scores.

To assess participants’ attitudes towards influenza and the use of influenza vaccine during pregnancy, respondents were asked to indicate their level of agreement with a series of statements on a 5-point scale.

Participants expressed their current willingness to get vaccinated during pregnancy on a 5-point likelihood scale. Subsequently, women were presented with 10 hypothetical scenarios and asked to indicate whether each of these scenarios would make them more or less likely than currently to be vaccinated during pregnancy. Overall willingness to vaccinate was calculated as the sum of willingness to vaccinate scores under all ten scenarios.

### Influenza vaccine uptake

The main outcome of this study was self-reported influenza vaccination status during current pregnancy. We classified women who were unsure whether they had been vaccinated as unvaccinated, and estimated the proportion of vaccinated participants with 95% exact binomial confidence intervals.

### Anchoring bias

In order to detect potential anchoring bias, we created four different questionnaire forms. In one pair of forms, attitude questions on vaccine side effects were followed by questions on perceived vaccine effectiveness. In the willingness to vaccinate domain, negative scenarios were presented first. In a second pair of forms, the attitude domain asked questions on perceived vaccine effectiveness first, and negative scenarios were presented at the end of the willingness to vaccinate domain. Within each pair of forms, response options were ordered from most positive to most negative in one form, and in reverse order in the other. Upon consent, each participant was randomly directed to fill out one of the four different forms. We used χ^2^-tests to compare the distribution of responses to attitude and willingness to vaccinate questions across the different questionnaire forms.

### Factor analysis

In order to define underlying attitudinal dimensions, we conducted exploratory factor analysis on the items in the attitudes domain using the iterated principal factor method. We examined polychoric correlation matrices [19] of items assessing respondents’ attitudes and used the Kaiser-Meyer-Olkin test [20] of sampling adequacy to confirm that the data was suitable for factor analysis (threshold > 0.7). We used parallel analysis and scree plot inflection points to determine the number of factors to be extracted. We used oblique promax rotation, and considered factor loadings > 0.3 to be significant for inclusion of each item into a given factor. Factor scores were calculated using the regression-based method, and were standardized to a mean of zero and standard deviation of one. Predicted factor scores for individual participants were used as independent variables in multivariable regression analysis.

### Willingness to pay

We used the Mann-Whitney U-test to compare median willingness to pay for one dose of influenza vaccine during pregnancy between vaccinated and unvaccinated individuals.

### Factors associated with influenza vaccination during pregnancy

We identified predictors of influenza vaccination uptake during pregnancy through Poisson regression models with robust standard errors, using prevalence ratios (PRs) and related 95% confidence intervals (CIs) as measures of association [21–23]. More details on the rationale for choosing these specific models can be found in the Supplementary Material for this manuscript.

In univariable analysis, we regressed each independent variable against the outcome and considered for inclusion in multivariable models those variables for which there was moderate to strong evidence for an association (Wald test *p*-value < 0.2). We excluded three variables (*Received information about the influenza vaccine from a polyclinic doctor, television, or newspapers*), because the number of participants responding ‘Yes’ was very small (< 7) and none had been vaccinated, precluding estimation of a prevalence ratio. Since 95% of participants were married, marital status was excluded from this analysis. Being personally advised to get vaccinated during pregnancy was strongly associated with receiving information on the influenza vaccine (Pearson’s χ^2^: 256.5; *p*-value < 0.001). Thus, the latter variable was excluded from the analysis.

Within each conceptual domain, we determined the set of independent variables most strongly associated with vaccination status by sequentially entering into the model candidate variables identified in the univariable analysis using a forward stepwise approach, starting with the most strongly associated variable. We retained variables with a Wald test p-value < 0.1. In the next stage, we sequentially entered the variables retained in different domains into the same multivariable regression model, retaining variables with a Wald test p-value < 0.05. Each variable initially excluded based on the results of univariable regression was again included into the final model to assess whether it would significantly contribute to it. No variable was reinserted into the final model based on this assessment.

All analyses were performed using Stata 14 (Stata Corporation).

### Ethics approval

The study was approved by the SingHealth Centralised Institutional Review Board on August 8th, 2017 (reference number: 2017/2667).

## Results

### Study population

**Fig. 2:**
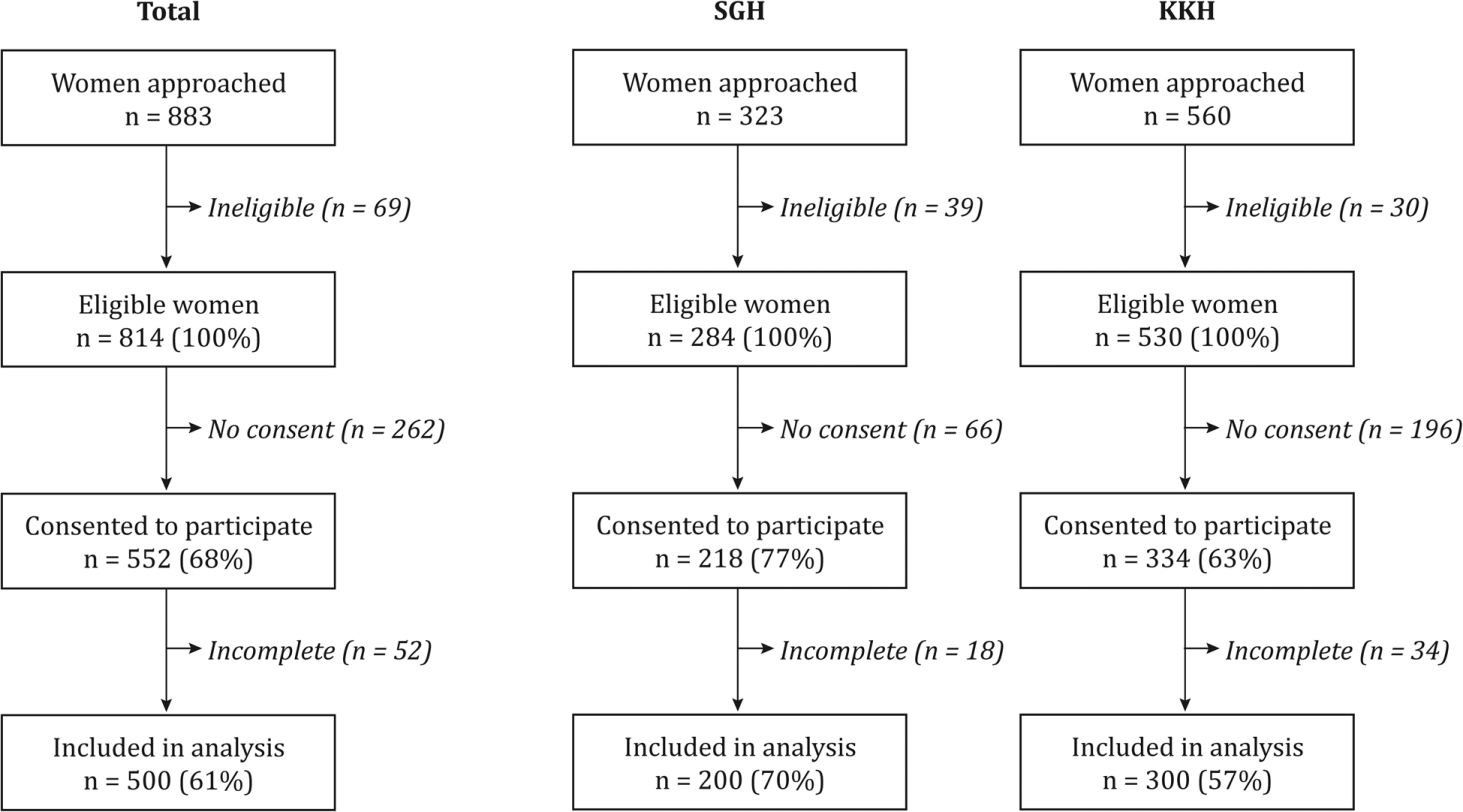
Recruitment chart. SGH: Singapore General Hospital; KKH: KK Women’s and Children Hospital

Of 883 women approached at the two study sites, two women aged < 21 years and 67 non-pregnant individuals were excluded, leaving 814 (92%) eligible women (Fig. 2). Of these, 552 (68%) agreed to participate and 500 (61%) submitted a completed questionnaire.

Most participants were Singaporean citizens (76%) or permanent residents (14%), and were predominantly of Chinese (40%), Malay (33%), or Indian (14%) ethnicity (Table 1). Respondents were comparable to the national population of women giving birth to live children in the same time period in terms of age, although women of Chinese ethnicity were under-represented and women of Malay ethnicity were over-represented in our sample. The majority of participants had a university degree (51%) or post-secondary education (37%) (Table 1).

**Table 1:** Socio-demographic characteristics of study participants and comparison with women giving birth to live children among Singapore population in 2017

	Study population		National population^a^
	n	%	%
Total	500	100	100
Age group (years)			
21 to 25 years	54	10.8	5.0
26 to 30 years	184	36.8	26.1
31 to 35 years	182	36.4	40.9
36 to 40 years	71	14.2	22.7
41 to 45 years	9	1.8	4.4
Trimester of pregnancy			
First (1 to 12 weeks)	49	9.8	
Second (13 to 27 weeks)	162	32.4	
Third (28 weeks and above)	289	57.8	
Residential status			
Citizen	378	75.6	
Permanent Resident	71	14.2	
Foreigner/ missing	51	10.2	
Ethnic group			
Chinese	200	40.0	58.3
Malay	163	32.6	20.1
Indian	68	13.6	9.7
Other^b^	50	10.0	11.9
Prefer not to answer	19	3.8	
Highest education level^c^			
Primary (PSLE or equivalent)	2	0.4	
Lower secondary	9	1.8	
Secondary (N/O levels pass)	49	9.8	
Post-secondary (A levels/ Nitec/higher Nitec/Master Nitec)	47	9.4	
Polytechnic	68	13.6	
Professional qualification and other Diploma (NIE/ITE/SIM diploma)	68	13.6	
University bachelor degree	204	40.8	
University Master’s degree and above	53	10.6	
Total monthly household income			
< $1000	14	2.8	
$1000–$4999	213	42.6	
$5000–$9999	127	25.4	
$10,000–$14,999	48	9.6	
$15,000–$19,999	10	2.0	
$20,000+	20	4.0	
Prefer not to answer	68	13.6	
Type of housing			
HDB^d^ 1–2 rooms	21	4.2	
HDB^d^ 3–4 rooms	274	54.8	
HDB^d^ 5 rooms	112	22.4	
Condominium	48	9.6	
Landed property	22	4.4	
Prefer not to answer	23	4.6	
Marital status			
Married	475	95.0	
Single, separated or divorced	11	2.2	
Prefer not to answer	14	2.8	
First pregnancy			
No	266	53.2	
Yes	234	46.8	
Eligible for Medisave			
No/ Not sure	79	15.8	
Yes	421	84.2	
Insurance^e^			
Yes	231	46.2	
No	127	25.4	
Not sure	142	28.4	

^a^ Data on women giving birth to live children extracted from Singapore Demographic Bulletin, 3rd Quarter 2017 from Singapore Immigration and Checkpoints Authority [24]

^b^ Includes Filipino, Caucasian, Javanese, Pakistani, Vietnamese, Myanmar, Arabic, Boyanese, Korean, Sikh, Asian

^c^ *PSLE* Primary School Leaving Examination, *N/O* Singapore General Certificate of Education: Normal/Ordinary Level, *GCE A* Singapore General Certificate of Education: Advanced Level, *Nitec* National Institute Of Technical Education Certificate, *NIE* National Institute of Education, *ITE* Institute of Technical Education, *SIM* Singapore Institute of Management

^d^ Singapore Housing and Development Board

^e^ Has private or employer health insurance that covers the cost of the influenza vaccine

### Influenza vaccine uptake

Self-reported influenza vaccine uptake during the current pregnancy was 9.8% (95% CI: 7.3–12.7%) (Table 2). There was no significant difference in vaccine uptake by trimester (Table 2). In addition, 35.2% (95% CI: 31.0–39.6) of participants reported being vaccinated against influenza at least once outside of pregnancy. Twenty-nine out of 500 participants (6%) were unsure whether they had been vaccinated against influenza during their current pregnancy and were classified as unvaccinated. Among 49 women vaccinated during their current pregnancy, about two thirds (63%) were vaccinated at a hospital, while the remainder were vaccinated at a private general practice or polyclinic. The most common reasons to get vaccinated against influenza during pregnancy were recommendation by a healthcare worker (57%), and protection from influenza in general (41%) (Additional file 2: Figure S1-A).

**Table 2:** Self-reported influenza vaccine uptake during current pregnancy by demographic group

Total	Total n	% vaccinated (95% CI)	
	500	9.8	(7.3; 12.7)
Age group (years)			
21 to 25	54	7.4	(2.1; 17.9)
26 to 30	184	14.7	(9.9; 20.6)
31 to 35	182	5.5	(2.7; 9.8)
36 to 46	80	10.0	(4.4; 18.8)
Trimester			
First (1 to 12 weeks)	49	4.1	(0.5; 14.0)
Second (13 to 27 weeks)	162	11.7	(7.2;17.7)
Third (28 weeks and above)	289	9.7	(6.5; 13.7)
Residential status			
Citizen	378	9.3	(6.5; 12.6)
Permanent resident	71	11.3	(5.0; 21.0)
Foreigner/*missing*	51	11.8	(4.4; 23.9)
Ethnicity			
Chinese	200	11.0	(7.0; 16.2)
Malay	163	6.1	(3.0; 11.0)
Indian	68	10.3	(4.2; 20.1)
Other^a^	50	18.0	(8.6; 31.4)
*Prefer not to answer*	19	5.3	(0.1; 26.0)
Education			
Secondary and below	60	3.3	(0.4; 11.5)
Post-secondary	183	6.0	(3.0; 10.5)
University Bachelor degree	204	13.2	(8.9; 18.7)
University Masters degree and above	53	17.0	(8.1; 29.8)
Income			
< $1000	14	7.1	(0.1; 33.9)
$1000–$4999	213	8.5	(5.1; 13.0)
$5000–$9999	127	10.2	(5.6; 16.9)
$10,000–$14,999	48	22.9	(12.0; 37.3)
$15,000–$19,999	10	10.0	(0.3; 44.5)
$20,000+	20	10.0	(1.2; 31.7)
*Prefer not to answer*	68	4.4	(0.9; 12.4)
Housing			
HDB^b^ 1–2 rooms	21	4.8	(0.1; 23.8)
HDB^b^ 3–4 rooms	274	6.6	(0.4; 10.2)
HDB^b^ 5 rooms	112	11.6	(6.3; 19.0)
Condo	48	12.5	(4.7; 25.2)
Landed property	22	45.5	(24.4; 67.8)
*Prefer not to answer*	23	4.3	(0.1; 21.9)
Marital status			
Married	475	9.3	(6.8; 12.2)
Single/separated/ divorced	11	36.4	(10.9; 69.2)
*Prefer not to answer*	14	7.1	(0.1; 33.9)
First pregnancy			
No	266	9.0	(5.9; 13.1)
Yes	234	10.7	(7.0; 15.4)
Eligible for Medisave			
No/ Not sure	79	8.9	(3.6; 17.4)
Yes	421	10.0	(7.3; 13.2)
Insurance^c^			
No	231	6.5	(3.7; 10.5)
Yes	127	22.1	(15.2; 30.3)
Not sure	142	4.2	(1.6; 9.0)

^a^ Includes Filipino, Caucasian, Javanese, Pakistani, Vietnamese, Myanmar, Arabic, Boyanese, Korean, Sikh, Asian

^b^ Singapore Housing and Development Board

^c^ Has private or employer health insurance that covers the cost of the influenza vaccine

Of 47 unvaccinated women in their first gestation trimester, 8 (17%) intended to get vaccinated against influenza during their current pregnancy. The proportion of women in their second and third trimester planning to get vaccinated during their current pregnancy was 9 and 3%, respectively. The main reasons not to get vaccinated included lack of recommendation (45%) and lack of information on the influenza vaccine (31%) (Additional file 2: Figure S1-B).

### Knowledge

Most participants (90%) identified viruses as the aetiological agent of influenza, although none recognised viruses as the sole cause. Overall, antibiotics were the most commonly selected option for influenza treatment (49%). The majority of participants (77%) were aware that there is an influenza vaccine. However, less than half (46%) knew that influenza vaccination is recommended during pregnancy.

### Attitudes

The majority of participants (> 56%) perceived themselves, their developing babies, and new-borns to be more vulnerable to severe influenza illness compared to other groups. Over half of all women (54%) felt that vaccination during pregnancy was effective in protecting them against influenza illness, but only a third of them (33%) believed it would help protect their new-born. Nearly half of all women (46%) felt more vulnerable to adverse events following vaccination during pregnancy, but only a small fraction of participants (21%) were worried about potential side effects on their developing baby.

Exploratory factor analysis on items in the attitude domain revealed two latent dimensions, labelled as “*Higher vaccine confidence*” and “*Higher perceived risk*” (Additional file 1: Table S3). The correlation between the two dimensions was 0.156. Based on factor loadings, the dimension *Higher vaccine confidence* was highly correlated with higher perceived vulnerability to severe influenza illness during gestation, the belief that vaccination during pregnancy is safe and effective in protecting both pregnant women and their new-borns from severe influenza illness, and concern about side effects on the developing baby. The dimension *Higher perceived risk* was correlated with high perceived vulnerability to severe influenza illness among pregnant mothers, developing babies, and new-borns, and fear of side effects of vaccination on both mother and developing baby. These two factors explained 83% of total variance in the attitudes data.

### Willingness to vaccinate

**Fig. 3:**
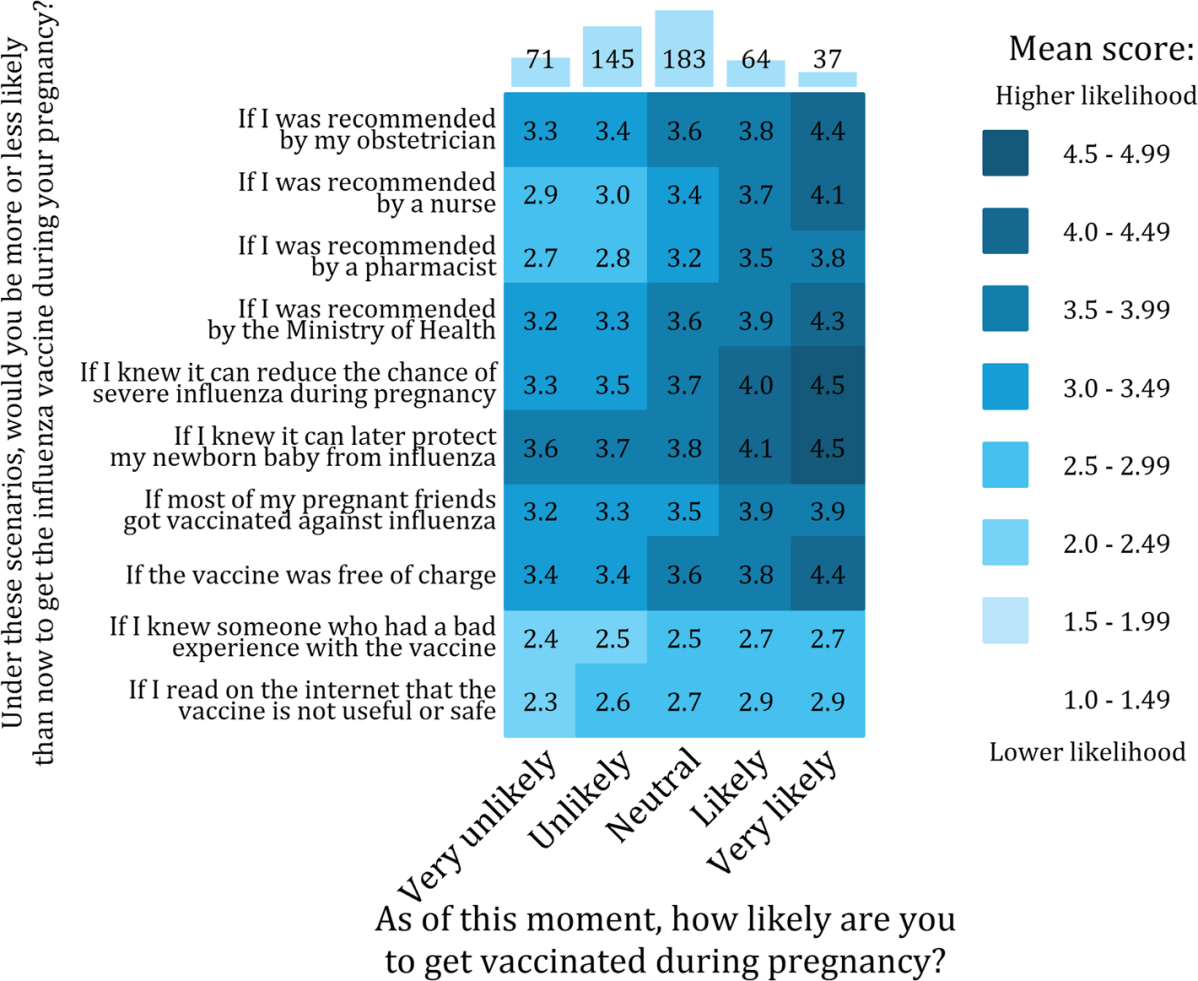
Willingness to vaccinate. Willingness to vaccinate under different scenarios (x-axis), by current willingness to vaccinate during pregnancy (y-axis). Darker colours indicate greater willingness than currently to vaccinate against influenza during pregnancy under the scenarios presented. Numbers in boxes represent respondent mean scores on a 5-point scale (1 = Very unlikely; 2 = Unlikely; 3 = Neutral; 4 = Likely; 5 = Very likely). The bar chart on the right denotes the distribution of respondents by category of current willingness to vaccinate

Overall, willingness to vaccinate against influenza was low (Fig. 3). Nearly half of unvaccinated women (48%) stated they were unlikely or very unlikely to get vaccinated during current gestation, and an additional 39% felt neutral about vaccination during pregnancy. A fraction of participants (14%) stated their willingness to vaccinate would not increase under any of the hypothetical scenarios presented. However, almost two thirds of women (> 60%) stated their willingness to vaccinate would increase if they knew that vaccination during pregnancy can protect them or their new-born from severe influenza illness. Other factors increasing participants’ willingness to vaccinate included recommendations from an obstetrician or the Ministry of Health, and free vaccination. Conversely, participants’ willingness to vaccinate was only moderately affected by recommendation from a pharmacist or a nurse (Fig. 3).

### Anchoring bias

There were no significant differences in the distribution of responses to questions across the four different questionnaire forms, indicating a negligible effect of question order or response option order on participants’ responses to either attitudes or willingness to vaccinate questions.

### Cues to vaccination

Among participants, 17% had received information about the influenza vaccine during their current pregnancy. The most common sources of information for these participants were friends, family or employers (37%), although women most commonly indicated their obstetrician (71%) and official websites (40%), such as the Ministry of Health or Health Promotion Board websites, as their preferred sources of information.

Only 12% of respondents had been personally advised to get vaccinated. Of these, 34% were recommended by their obstetrician, while others were advised by their general practitioner (22%), a nurse (22%), or friends and family (21%).

### Willingness to pay

Out of 421 participants eligible for Medisave, 34 (8%) stated they would not pay any amount for one dose of influenza vaccine during pregnancy. Among those willing to pay, median willingness to pay for one dose out of Medisave was SGD50 (~USD37) (range: 1–600 SGD/ 1–440 USD; interquartile range: SGD50–100/ USD37–74). This was higher than the price of the vaccine in most clinics and general practices in Singapore, and higher than median willingness to pay among parents of children in the age groups recommended for vaccination [17]. There was no significant difference in median willingness to pay out of Medisave between vaccinated and unvaccinated women (SGD60 vs SGD50, Mann-Whitney U test: *p*-value = 0.9).

### Factors associated with influenza vaccination

Results from univariable analysis are reported in Additional file 1: Tables S4 and S5. In the final multivariable analysis, women who were personally advised to receive the influenza vaccine during pregnancy were seven times more likely to be vaccinated compared to women who were not recommended vaccination (PR = 7.11; 95% CI: 3.92–12.90) (Table 3). Other variables independently associated with higher vaccination uptake included vaccination during a previous pregnancy (PR = 2.51; 95% CI: 1.54–4.11), and having private or employer health insurance that covers the cost of the vaccine (PR = 2.32; 95% CI: 1.43–3.76). There was a linear association between vaccination uptake and the *Higher vaccine confidence* factor, with a 62% increase in vaccination prevalence per 1 standard deviation increase in the score for this factor (PR = 1.62; 95% CI: 1.30–2.01) (Table 3).

**Table 3:** Multivariable analysis of factors associated with reported influenza vaccination uptake during current pregnancy

	Multivariable analysis		
	Adjusted PR^a^	95% CI	*P*-value
Cues to vaccination			
Was personally advised to get vaccinated against influenza during current pregnancy			< 0.001
No	1		
Yes	7.11	3.92; 12.90	
Not sure	1.98	0.71; 5.49	
Vaccination practices			
Was vaccinated against influenza during previous pregnancy			
No^b^	1		
Yes	2.51	1.54; 4.11	< 0.001
Insurance status			0.001
Not insured	1		
Insured	2.32	1.43; 3.76	
Not sure	1.03	0.46; 2.26	
*Higher vaccine confidence* (Factor 1)^c,d^	1.62^c^	1.30; 2.01	< 0.001
Willingness to vaccinate^c^	1.06	1.01; 1.12	0.011

^a^ Adjusted prevalence ratio

^b^ Includes those who did not get vaccinated during previous pregnancy, those unsure whether they got vaccinated during previous pregnancy, and those who were never pregnant before their current pregnancy

^c^ Linear trend

^d^ For variable composition of the attitude dimension *Higher vaccine confidence,* see Table S3

### Other vaccines

Few participants reported receiving the tetanus (2%) or combined tetanus-diphtheria-pertussis vaccine (3%) during pregnancy. Uptake of vaccination against Hepatitis A and B was 2 and 6%, respectively, while < 1% of participants reported receiving meningococcal vaccination.

## Discussion

### Main findings

In this survey, we assessed the uptake and determinants of influenza vaccination among pregnant women in Singapore. Although most participants were knowledgeable about influenza, a few common misconceptions were identified, such as the belief that influenza can be treated with antibiotics. Although awareness of the influenza vaccine was high, and the majority of women had a positive attitude towards vaccination during pregnancy, willingness to vaccinate was low. Only 10% of participants reported being vaccinated against influenza during their current gestation. Being personally advised to get vaccinated during pregnancy was the most significant factor associated with increased vaccination uptake, but only a small proportion of women were personally advised to get vaccinated.

Studies from other Southeast Asian countries reported equally low (< 10%) influenza vaccine uptake [25–28]. In Hong Kong, women were six times more likely to get vaccinated during pregnancy if recommended by a healthcare professional [26, 27]. Similarly, recommendation from a healthcare provider was associated with higher willingness to vaccinate among pregnant women in Thailand [28]. Even in countries where influenza vaccine uptake among pregnant women is comparatively high, recommendation from healthcare professionals was consistently one of the most important factors increasing vaccination uptake [29–31]. In our study, women who were personally advised to be vaccinated against influenza during pregnancy were seven times more likely to take up the vaccine. However, only a few pregnant women were personally advised or informed about the influenza vaccine, and less than half of participants were aware of existing recommendations. In some settings, pharmacist-led provision of influenza vaccines is well accepted [32, 33], and contributes to increased vaccine uptake [33–35]. However, participants’ willingness to vaccinate was only moderately affected by recommendation from a pharmacist in our study. This finding highlights the importance of investigating factors which may affect the effectiveness of maternal vaccination strategies in different cultural contexts.

Healthcare providers who are more knowledgeable [36, 37] and confident [38, 39] about the influenza vaccine are more likely to recommend vaccination and obtain high coverage among expecting mothers. In Singapore, qualitative studies have revealed limited influenza vaccine acceptance among healthcare professionals [40, 41]. Provider-targeted interventions involving education, automatic reminders, and vaccination training effectively increased vaccination uptake among pregnant patients in other settings [42], and may be key to increasing uptake among pregnant women and other high-risk groups [17] in Singapore. Lack of incentives and financial barriers, including the cost of ordering and stocking the vaccine, may also influence healthcare providers’ practices [43, 44].

Consistent with previous findings [27, 31, 36, 45–47], we found that greater concern about influenza illness coupled with greater confidence in influenza vaccine were associated with increased vaccination uptake during pregnancy. In contrast, greater perceived risk of influenza severity and vaccine side effects did not significantly affect vaccination uptake in this population. In contrast, concerns about vaccine side effects have been found to be negatively associated with vaccine uptake during pregnancy in other settings [46, 48].

Among our study participants, lack of information on the influenza vaccine was one of the most common reasons not to get vaccinated. This was in agreement with a recent survey among parents of young children in Singapore, where those who felt well-informed were more likely to have vaccinated their child in the past [17]. Patient-targeted educational interventions are moderately effective in increasing influenza vaccine uptake [42], but may help in curbing common misconceptions, such as the belief that influenza can be treated with antibiotics.

### Limitations

Our study sample only included participants from public hospitals, and may not have been representative of all pregnant women in Singapore. However, both hospitals catered to patients receiving public and private healthcare. Furthermore, both hospitals are responsible for antenatal care covering more than a third of the annual birth cohort in Singapore. Women who got vaccinated against influenza during their pregnancy may have been more likely to participate in this survey, resulting in an overestimation of vaccine coverage. Given the low reported uptake, this potential bias may be negligible. If participants who were planning to get vaccinated against influenza at the time of the survey actually did so at a later stage of their pregnancy, our survey may have underestimated vaccine coverage in this population. However, there was no evidence of increasing uptake by trimester of pregnancy. Vaccination status was self-reported and thus potentially subject to poor recall. Currently, there is no adult immunization registry in Singapore, and our survey was anonymous so we did not have access to medical records to validate self-reported vaccination status. However, previous studies have shown that self-reported influenza vaccination uptake overestimates coverage as reported in computerized vaccination registries [49]. In our study, < 6% of women were unsure of their influenza vaccination status. Assuming all these women were in fact vaccinated, though unrealistic, would still only increase coverage to about 16%. Given that personal recommendation was the key factor associated with vaccination, and that pregnant women tend to be more aware of their health and well-being, we expect that women who were vaccinated during their current pregnancy would have been likely to remember it. Excluding those who were unsure whether they had been vaccinated during current pregnancy from the multivariable analysis did not alter the results (data not shown). A number of vaccines are recommended during pregnancy, and respondents may not have been able to reliably distinguish between these. However, reported vaccination during pregnancy was low for all vaccines and influenza vaccine was the most commonly reported. Since the survey was anonymous, it was not possible to ensure that the same woman did not participate on more than one occasion. However, no remuneration was given to participants for taking part in this survey, limiting the incentive for women to participate more than once. In addition, the survey was conducted within a time span of less than two months, so that a woman would likely remember if she had taken part in the survey before. Finally, the low number of vaccinated individuals may have limited study power to detect additional factors associated with vaccination.

## Conclusions

Coverage of influenza vaccine among pregnant women is low in Singapore. Encouraging healthcare professionals, especially obstetricians, to recommend influenza vaccination is key to improving vaccine uptake in this population. In contrast to other settings, the potential impact of pharmacy-led maternal influenza vaccination strategies may be limited in Singapore. Public communication about influenza vaccination should target common misconceptions about influenza and influenza vaccines. The baseline data collected in this survey can be used to measure the impact of future interventions on influenza vaccine coverage among pregnant women. With the recent inclusion of maternal pertussis vaccination into the pregnancy immunisation schedule and progress towards novel respiratory syncytial virus vaccines, the repertoire of vaccines recommended in pregnancy is likely to increase. Our findings provide additional insights for public health authorities to plan and implement targeted strategies to improve coverage of maternal vaccinations.

## Additional files

Additional file 1: **Table S1.** Details of survey questionnaire. **Table S2.** Scoring of knowledge questions. **Table S3.** Exploratory factor analysis. **Table S4.** Univariable analysis of socio-demographic variables, practices, and cues to vaccination. **Table S5.** Univariable analysis of knowledge, attitude, and willingness to vaccinate variables (DOCX 290 kb)

Additional file 2: **Figure S1.** Figure showing reasons for taking and not taking influenza vaccine during current pregnancy among study participants. (TIFF 5357 kb)

## Data Availability

The datasets used and/or analysed during the current study are available from the corresponding author on reasonable request.

## Abbreviations

95% CI: 95% Confidence Interval
GCE A: Singapore General Certificate of Education: Advanced Level
GCE N/O: Singapore General Certificate of Education: Normal/Ordinary Level
HDB: Singapore Housing and Development Board
ITE: Institute of Technical Education
KKH: KK Women’s and Children Hospital Singapore
NIE: National Institute of Education
Nitec: National Institute of Technical Education Certificate
OBGYN: Obstetrics and Gynaecology
PR: Prevalence Ratio
PSLE: Primary School Leaving Examination
SGD: Singapore Dollar
SGH: Singapore General Hospital
SIM: Singapore Institute of Management
USD: United States Dollar
