# Supplementary material for: Coverage and determinants of influenza vaccine among pregnant women: a cross-sectional study
Source: BMC Public Health. 2019 Jul 5;19:890. doi: 10.1186/s12889-019-7172-8 (PMC6612156; doi:10.1186/s12889-019-7172-8)
Supplement: Supplementary file 1 — Table S1. Details of survey questionnaire. Table S2. Scoring of knowledge questions. Table S3. Exploratory factor analysis. Table S4. Univariable analysis of socio-demographic variables, practices, and cues to vaccination. Table S5. Univariable analysis of knowledge, attitude, and willingness to vaccinate variables (DOCX 290 kb) [file 12889_2019_7172_MOESM1_ESM.docx]

Additional material for:

**Coverage and determinants of influenza vaccine among pregnant women: a cross-sectional study**

Vittoria Offeddu^a#^, Clarence C Tam^a,b#^, Tze Tein Yong^c^, Lay Kok Tan^c^, Koh Cheng Thoon^d^, Nicole Lee^d^, Thiam Chye Tan^d^,George SH Yeo^d^, Chee Fu Yung^d,e^*

^a^ Saw Swee Hock School of Public Health, National University of Singapore and National University Health System, 117549 Singapore; ^b^ London School of Hygiene & Tropical Medicine, WC1E 7HT London, United Kingdom; ^c^ Singapore General Hospital, 169608 Singapore; ^d^ KK Women's and Children's Hospital, 229899 Singapore; ^e^ Lee Kong Chian School of Medicine, NTU Imperial College

Content

[1. Supplementary Figure 2](#_Toc4748622)

[Figure S1. Reasons for taking (A, n = 49) and not taking (B, n = 451) influenza vaccine during current pregnancy 2](#_Toc4748623)

[2. Supplementary Tables 3](#_Toc4748624)

[Table S1. Details of survey questionnaire 3](#_Toc4748625)

[Table S2. Scoring of knowledge questions 4](#_Toc4748626)

[Table S3. Exploratory factor analysis 5](#_Toc4748627)

[Table S4. Univariable analysis of socio-demographic variables, practices, and cues to vaccination 6](#_Toc4748628)

[Table S5. Univariable analysis of knowledge, attitude, and willingness to vaccinate variables 11](#_Toc4748629)

[3. Supplementary information: link function for Poisson regression model 12](#_Toc4748630)

# **Supplementary Figure**

## **Figure S1.** Reasons for taking (A, n = 49) and not taking (B, n = 451) influenza vaccine during current pregnancy

HCW = healthcare worker; Flu = influenza


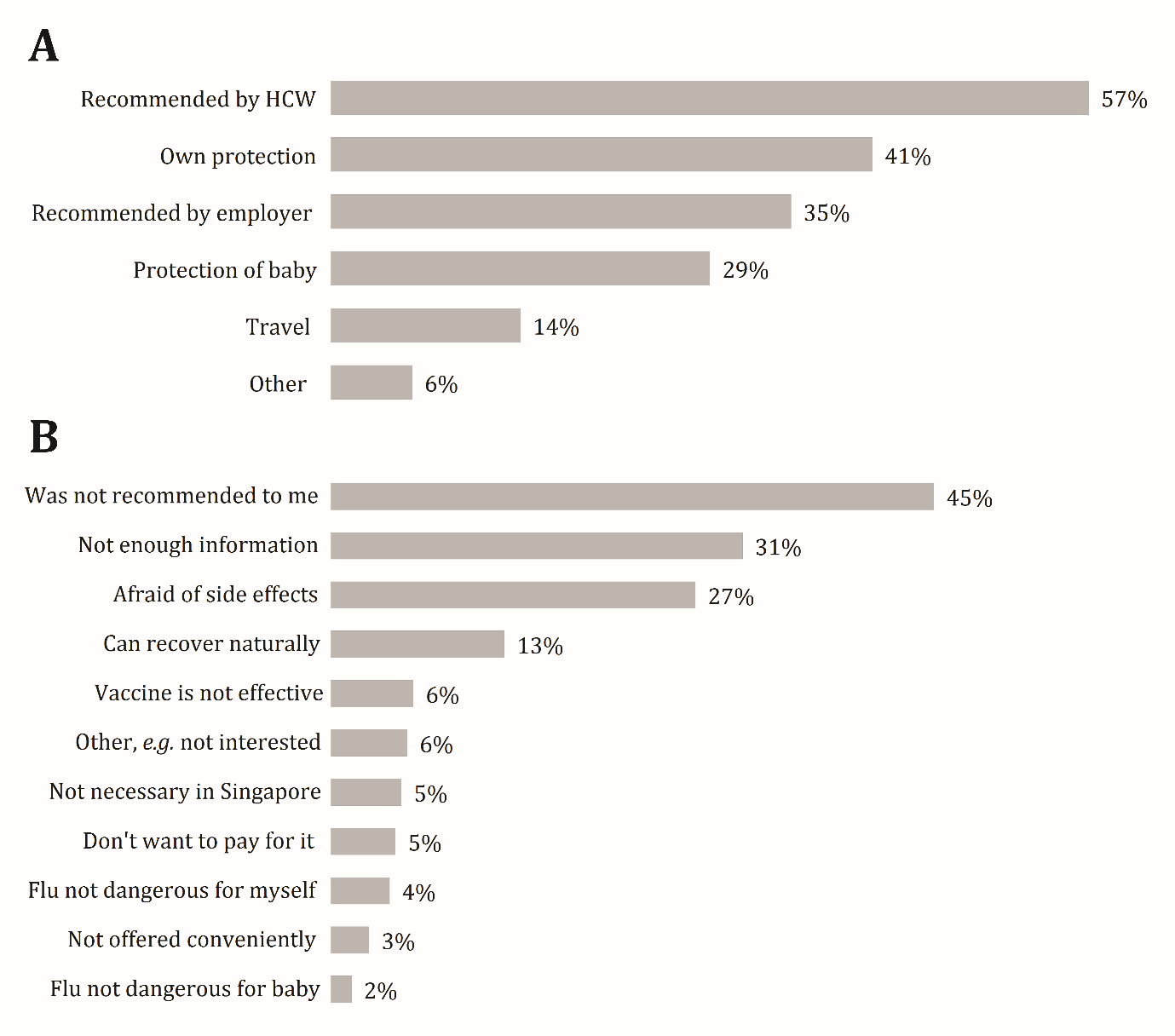


# Supplementary Tables

## Table S1. Details of survey questionnaire

| **Domain** | **Variables assessed** |
| --- | --- |
| Demographics | Age; trimester of pregnancy; residential status; ethnicity; highest education level; total monthly household income; type of housing; marital status. |
| Knowledge | Knowledge of influenza and influenza vaccine. |
| Attitudes | Perceived susceptibility to influenza; perceived benefits of the influenza vaccine; perceived barriers to vaccination during pregnancy. |
| Insurance status | Eligibility for Medisave; possession of private or employer health insurance that covers the cost of the influenza vaccine. |
| Willingness to pay | Willingness to pay for one dose of the influenza vaccine during pregnancy; participants were allowed to state any amount, including zero. |
| Vaccination history | Vaccination history during previous pregnancy; vaccination history outside of pregnancy; reasons to vaccinate during pregnancy; reasons not to get vaccinated during pregnancy. |
| Cues to vaccination | External cues to vaccination during pregnancy, including information and advice sources. |
| Willingness to vaccinate | Current and hypothetical willingness to vaccinate against influenza during pregnancy under different proposed scenarios. |

## Table S2. Scoring of knowledge questions

| **Knowledge of influenza** (multiple choice) | | | | | |
| --- | --- | --- | --- | --- | --- |
| **Item** | **Answer options** | **Score** | | | |
|  |  | **If selected** | | **If not selected** | |
| *Which of these are common symptoms of influenza?* | Fever | 1 | | 0 | |
|  | Diarrhoea | 0 | | 1 | |
|  | Vomiting | 0 | | 1 | |
|  | Sore throat | 1 | | 0 | |
|  | Cough | 1 | | 0 | |
|  | Chest pain | 0 | | 1 | |
|  | Rash | 0 | | 1 | |
|  | Muscle pain | 1 | | 0 | |
| *Which of the following causes influenza?* | Viruses | 1 | | 0 | |
|  | Bacteria | 0 | | 1 | |
|  | Cold weather | 0 | | 1 | |
|  | Mosquito bites | 0 | | 1 | |
|  | Not sure | 0 | | 1 | |
|  | Others (please specify) | 0 | | 1 | |
| *Influenza can be treated with:* | Antibiotics, e.g. Augmentin, Zithromax | 0 | | 1 | |
|  | Fever medications, e.g. Aspirin, paracetamol | 1 | | 0 | |
|  | Antivirals, e.g. Relenza, Tamiflu | 1 | | 0 | |
|  | Pain medications, e.g. Ibuprofen, Nurofen | 1 | | 0 | |
|  | Not sure | 0 | | 1 | |
|  | Others (please specify) | 0 | | 1 | |
| **Knowledge of influenza vaccine** (single choice) | | | | | |
| **Item** | | | **Answer options** | | **Score** |
| *Before today, did you know that there is a vaccine against influenza?* | | | Yes | | 1 |
|  | | | No | | 0 |
|  | | | Not sure | | 0 |
| *The influenza vaccine is only necessary when you travel overseas in winter.* | | | Agree | | 0 |
|  | | | Disagree | | 1 |
|  | | | Not sure | | 0 |
| *The Ministry of Health recommends that pregnant women should be vaccinated against influenza.* | | | Agree | | 1 |
|  | | | Disagree | | 0 |
|  | | | Not sure | | 0 |

## **Table S3. Exploratory factor analysis**

Factor loadings and communalities based on an iterated principal factor analysis with oblique promax rotation for eight items contained in the attitudes domain. Initial Eigenvalues for Factor 1 and Factor 2 were 2.36 and 1.27, respectively.

| **Item** | **Factor loading** | | **Communality** |
| --- | --- | --- | --- |
|  | **Factor1:**  ***Higher vaccine confidence*** | **Factor 2:**  ***Higher perceived risk*** |  |
| While pregnant, I am more vulnerable to severe influenza illness | **0.36** | **0.47** | 0.41 |
| Getting vaccinated against influenza while pregnant can be harmful for my baby | **-0.67** | **0.37** | 0.51 |
| The influenza vaccine is safe for pregnant women. | **0.78** | 0.01 | 0.61 |
| The influenza vaccine is effective in protecting me against influenza during pregnancy | **0.74** | 0.19 | 0.63 |
| Getting the influenza vaccine during pregnancy can help protect my newborn baby from influenza for several months after birth | **0.57** | 0.19 | 0.38 |
| If I get influenza while pregnant, my baby could be affected | 0.07 | **0.60** | 0.38 |
| Compared to other age groups, newborn babies are more vulnerable to severe influenza illness | 0.15 | **0.53** | 0.33 |
| During pregnancy, I am more vulnerable to the side effects from the influenza vaccine | -0.29 | **0.60** | 0.39 |

## **Table S4. Univariable analysis of socio-demographic variables, practices, and cues to vaccination**

Descriptive analysis of associations of socio-demographic characteristics, practices and cues to vaccination with self-reported influenza vaccination uptake during current pregnancy. Of 500 participants, 49 reported being vaccinated during current pregnancy.

|  | | **Total** | **Vaccinated** | | **Univariable analysis** | | |
| --- | --- | --- | --- | --- | --- | --- | --- |
|  | | **n** | **n** | **%** | **PR^a^** | **95% CI** | **P-value** |
| **Demographics** | |  |  |  |  |  |  |
| Age group (years) | |  |  |  |  |  | 0.038 |
|  | 21 to 25 | 54 | 4 | 7.4 | 1 |  |  |
|  | 26 to 30 | 184 | 27 | 14.7 | 1.98 | 0.72; 5.42 |  |
|  | 31 to 35 | 182 | 10 | 5.5 | 0.74 | 0.24; 2.27 |  |
|  | 36 to 46 | 80 | 8 | 10.0 | 1.35 | 0.43; 4.27 |  |
| Trimester | |  |  |  |  |  | 0.326 |
|  | First (1 to 12 weeks) | 49 | 2 | 4.1 | 1 |  |  |
|  | Second (13 to 27 weeks) | 162 | 19 | 11.7 | 2.87 | 0.69; 11.92 |  |
|  | Third (28 weeks and above) | 289 | 28 | 9.7 | 2.37 | 0.58; 9.66 |  |
| Residential status | |  |  |  |  |  | 0.770 |
|  | Citizen | 378 | 35 | 9.3 | 1 |  |  |
|  | Permanent resident | 71 | 8 | 11.3 | 1.22 | 0.59; 2.51 |  |
|  | Foreigner/*missing* | 51 | 6 | 11.8 | 1.27 | 0.56; 2.87 |  |
| Ethnicity | |  |  |  |  |  | 0.146 |
|  | Chinese | 200 | 22 | 11.0 | 1 |  |  |
|  | Malay | 163 | 10 | 6.1 | 0.56 | 0.27; 1.14 |  |
|  | Indian | 68 | 7 | 10.3 | 0.94 | 0.42; 2.09 |  |
|  | Other^b^ | 50 | 9 | 18.0 | 1.64 | 0.80; 3.33 |  |
|  | *Prefer not to answer* | 19 | 1 | 5.3 | 0.48 | 0.07; 3.36 |  |
| Education | |  |  |  |  |  | 0.016 |
|  | Secondary and below | 60 | 2 | 3.3 | 1 |  |  |
|  | Post-secondary | 183 | 11 | 6.0 | 1.80 | 0.41; 7.92 |  |
|  | University Bachelor degree | 204 | 27 | 13.2 | 3.97 | 0.97; 16.24 |  |
|  | University Masters degree and above | 53 | 9 | 17.0 | 5.09 | 1.15; 22.57 |  |
| Income | |  |  |  |  |  | 0.063 |
|  | <$1000 | 14 | 1 | 7.1 | 1 |  |  |
|  | $1000-$4999 | 213 | 18 | 8.5 | 1.18 | 0.17; 8.25 |  |
|  | $5000-$9999 | 127 | 13 | 10.2 | 1.43 | 0.20; 10.17 |  |
|  | $10,000-$14,999 | 48 | 11 | 22.9 | 3.20 | 0.45; 22.79 |  |
|  | $15,000-$19,999 | 10 | 1 | 10.0 | 1.40 | 0.10; 19.88 |  |
|  | $20,000+ | 20 | 2 | 10.0 | 1.40 | 0.14; 14.01 |  |
|  | *Prefer not to answer* | 68 | 3 | 4.4 | 0.62 | 0.07; 5.52 |  |
| Housing | |  |  |  |  |  | <0.001 |
|  | HDB^c^ 1-2 rooms | 21 | 1 | 4.8 | 1 |  |  |
|  | HDB^c^ 3-4 rooms | 274 | 18 | 6.6 | 1.38 | 0.19; 9.85 |  |
|  | HDB^c^ 5 rooms | 112 | 13 | 11.6 | 2.44 | 0.34; 17.69 |  |
|  | Condo | 48 | 6 | 12.5 | 2.63 | 0.36; 20.51 |  |
|  | Landed property | 22 | 10 | 45.5 | 9.55 | 1.33; 68.36 |  |
|  | *Prefer not to answer* | 23 | 1 | 4.4 | 0.91 | 0.06; 13.73 |  |
| Marital status | |  |  |  |  |  | 0.005 |
|  | Married | 475 | 44 | 9.3 | 1 |  |  |
|  | Single/separated/ divorced | 11 | 4 | 36.4 | 3.93 | 1.71; 9.02 |  |
|  | *Prefer not to answer* | 14 | 1 | 7.1 | 0.77 | 0.11; 5.21 |  |
| First pregnancy | |  |  |  |  |  |  |
|  | No | 266 | 24 | 9.0 | 1 |  |  |
|  | Yes | 234 | 25 | 10.7 | 1.18 | 0.70; 2.02 | 0.534 |
|  | |  |  |  |  |  |  |
| **Cues to vaccination** | |  |  |  |  |  |  |
| Received information about the influenza vaccine during current pregnancy (any source) | |  |  |  |  |  | <0.001 |
|  | No | 368 | 9 | 2.5 | 1 |  |  |
|  | Yes | 86 | 38 | 44.2 | 18.07 | 9.08; 35.96 |  |
|  | Not sure | 46 | 2 | 4.4 | 1.78 | 0.40; 7.99 |  |
| Received information about the influenza vaccine from an obstetrician | |  |  |  |  |  |  |
|  | No | 477 | 34 | 7.1 | 1 |  |  |
|  | Yes | 23 | 15 | 65.2 | 9.15 | 5.89; 14.22 | <0.001 |
| Received information about the influenza vaccine from a private general practitioner | |  |  |  |  |  |  |
|  | No | 487 | 42 | 8.6 | 1 |  |  |
|  | Yes | 13 | 7 | 53.9 | 6.24 | 3.49; 11.16 | <0.001 |
| Received information about the influenza vaccine from a nurse | |  |  |  |  |  |  |
|  | No | 476 | 41 | 8.6 | 1 |  |  |
|  | Yes | 24 | 8 | 33.3 | 3.87 | 2.05; 7.32 | <0.001 |
| Received information about the influenza vaccine from official websites, *e.g.* Ministry of Health, Health Promotion Board, or World Health Organization | |  |  |  |  |  |  |
|  | No | 487 | 44 | 9.0 | 1 |  |  |
|  | Yes | 13 | 5 | 38.5 | 4.26 | 2.02; 8.96 | <0.001 |
| Received information about the influenza vaccine from the internet, including social media like facebook or twitter | |  |  |  |  |  |  |
|  | No | 491 | 45 | 9.2 | 1 |  |  |
|  | Yes | 9 | 4 | 44.4 | 4.85 | 2.22; 10.61 | <0.001 |
| Received information about the influenza vaccine from other sources, e.g. friends, family, or employer | |  |  |  |  |  |  |
|  | No | 468 | 32 | 6.8 | 1 |  |  |
|  | Yes | 32 | 17 | 53.1 | 7.77 | 4.87; 12.40 | <0.001 |
| Was personally advised to get vaccinated against influenza during current pregnancy (any source) | |  |  |  |  |  | <0.001 |
|  | No | 394 | 12 | 3.1 | 1 |  |  |
|  | Yes | 58 | 34 | 58.6 | 19.25 | 10.58; 35.01 |  |
|  | Not sure | 48 | 3 | 6.3 | 2.05 | 0.60; 7.02 |  |
| Was personally advised by an obstetrician | |  |  |  |  |  |  |
|  | No | 478 | 34 | 7.1 | 1 |  |  |
|  | Yes | 22 | 15 | 68.2 | 9.59 | 6.22; 14.77 | <0.001 |
| Was personally advised by a private general practitioner | |  |  |  |  |  |  |
|  | No | 487 | 44 | 9.0 | 1 |  |  |
|  | Yes | 13 | 5 | 38.5 | 4.26 | 2.02; 8.96 | <0.001 |
| Was personally advised by a polyclinic doctor | |  |  |  |  |  |  |
|  | No | 494 | 46 | 9.3 | 1 |  |  |
|  | Yes | 6 | 3 | 50.0 | 5.37 | 2.30; 12.53 | <0.001 |
| Was personally advised by a pharmacist | |  |  |  |  |  |  |
|  | No | 498 | 48 | 9.6 | 1 |  |  |
|  | Yes | 2 | 1 | 50.0 | 5.19 | 1.26; 21.32 | 0.022 |
| Was personally advised by a nurse | |  |  |  |  |  |  |
|  | No | 487 | 42 | 8.6 | 1 |  |  |
|  | Yes | 13 | 7 | 53.9 | 6.24 | 3.49; 11.16 | <0.001 |
| Was personally advised by friends and family | |  |  |  |  |  |  |
|  | No | 488 | 43 | 8.8 | 1 |  |  |
|  | Yes | 12 | 6 | 50.0 | 5.67 | 3.01; 10.70 | <0.001 |
| Was personally advised by other sources^d^ | |  |  |  |  |  |  |
|  | No | 491 | 42 | 8.6 | 1 |  |  |
|  | Yes | 9 | 7 | 77.8 | 9.09 | 5.78; 14.32 | <0.001 |
|  |  |  |  |  |  |  |  |
| **Insurance status** | |  |  |  |  |  |  |
| Eligible for Medisave | |  |  |  |  |  |  |
|  | No/ not sure | 79 | 7 | 8.9 | 1 |  |  |
|  | Yes | 421 | 42 | 10.0 | 1.13 | 0.52; 2.42 | 0.761 |
| Has private or employer health insurance that covers the cost of the influenza vaccine | |  |  |  |  |  | <0.001 |
|  | No | 231 | 15 | 6.5 | 1 |  |  |
|  | Yes | 127 | 28 | 22.1 | 3.40 | 1.88; 6.12 |  |
|  | Not sure | 142 | 6 | 4.2 | 0.65 | 0.26; 1.64 |  |
|  | |  |  |  |  |  |  |
| **Vaccination practices** | |  |  |  |  |  |  |
| Vaccinated against influenza during previous pregnancy^e^ | |  |  |  |  |  | <0.001 |
|  | No | 226 | 11 | 4.9 | 1 |  |  |
|  | Yes | 20 | 11 | 55.0 | 11.30 | 5.61; 22.78 |  |
|  | Not sure | 20 | 2 | 10.0 | 2.05 | 0.49; 8.66 |  |
| Ever vaccinated against influenza outside of pregnancy | |  |  |  |  |  | 0.002 |
|  | No | 260 | 15 | 5.8 | 1 |  |  |
|  | Yes | 176 | 29 | 16.5 | 2.86 | 1.58; 5.17 |  |
|  | Not sure | 64 | 5 | 7.8 | 1.35 | 0.51; 3.59 |  |
| Received at least one other vaccine during current pregnancy^f^ | |  |  |  |  |  |  |
|  | No | 456 | 33 | 7.2 | 1 |  |  |
|  | Yes | 44 | 16 | 36.4 | 5.02 | 3.01; 8.38 | <0.001 |

^a^ Unadjusted prevalence ratio

^b^ Includes Filipino, Caucasian, Javanese, Pakistani, Vietnamese, Myanmar, Arabic, Boyanese, Korean, Sikh, Asian

^c^ Singapore Housing and Development Board

^d^ Includes employer (n = 7), colleague (n = 1), and myself (n = 1)

^e^ Among women who have been pregnant previously (n = 24)

^f^ Includes combined Tetanus-Diphteria-Pertussis, Tetanus, Hepatitis A, Hepatitis B, and Meningococcal.

## **Table S5. Univariable analysis of knowledge, attitude, and willingness to vaccinate variables**

Descriptive analysis of relationship between self-reported vaccination status during current pregnancy and mean score for knowledge, attitudes, and willingness to vaccinate. Of 500 participants, 49 reported being vaccinated during current pregnancy.

|  | | **Univariable analysis** | | |
| --- | --- | --- | --- | --- |
|  | | **PR^a^** | **95% CI** | **P-value** |
| **Knowledge level^b^** | | 1.30 | 1.18; 1.44 | <0.001 |
|  | |  |  |  |
| **Attitudes** | |  |  |  |
| *Perceived susceptibility*^c^ | |  |  |  |
|  | While pregnant, I am more vulnerable to severe influenza illness. | 1.91 | 1.29; 2.84 | 0.001 |
|  | If I get influenza while pregnant, my baby could be affected. | 1.40 | 0.89; 2.20 | 0.150 |
|  | Compared to other age groups, newborn babies are more vulnerable to severe influenza illness. | 1.32 | 0.80; 2.17 | 0.279 |
| *Perceived benefits of vaccination*^c^ | |  |  |  |
|  | The influenza vaccine is effective in protecting me against influenza during pregnancy. | 3.12 | 2.05; 4.76 | <0.001 |
|  | Getting the influenza vaccine during pregnancy can help protect my newborn baby from influenza for several months after birth. | 2.27 | 1.55; 3.32 | <0.001 |
|  | The influenza vaccine is safe for pregnant women. | 3.60 | 2.71; 4.79 | <0.001 |
| *Perceived barriers to vaccination*^c^ | |  |  |  |
|  | During pregnancy, I am more vulnerable to the side effects from the influenza vaccine. | 0.46 | 0.32; 0.66 | <0.001 |
|  | Getting vaccinated against influenza while pregnant can be harmful for my baby. | 0.32 | 0.22; 0.46 | <0.001 |
| *Higher vaccine confidence (Factor 1)*^b,d^ | | 2.77 | 2.34; 3.27 | <0.001 |
| *Higher perceived risk (Factor 2)*^b,d^ | | 0.99 | 0.70; 1.41 | 0.974 |
|  |  |  |  |  |
| **Willingness to vaccinate**^a^ | | 1.20 | 1.15; 1.26 | <0.001 |

^a^ Unadjusted prevalence ratio

^b^ Linear trend

^c^ Linear trend on a 5-point scale (1 = Strongly disagree, 2 = Disagree, 3 = Neutral, 4 = Agree, 5 = Strongly agree)

^d^ For factor loadings on individual items, see Table S3

# **Supplementary information: link function for Poisson regression model**

In the specific context of vaccination, what matters from an epidemiological and public health perspective is vaccination coverage, or the prevalence of vaccination. The prevalence ratio (PR) gives an indication of the factor by which vaccination coverage changes in relation to changes in explanatory variables. In this context, the interpretation of PRs is more intuitive than the interpretation of odds ratios (ORs) obtained through logistic regression models, which are more commonly used for binary outcomes. In particular, as vaccination coverage increases, the odds of vaccination provide an increasingly poor estimate of vaccination prevalence, and as vaccination coverage increases in one group compared with another, the OR becomes a less reliable estimate of the PR. This is misleading for policy makers, who may inappropriately interpret ORs and PRs as being interchangeable.

In this manuscript, we chose a Poisson model with robust errors as the appropriate analysis model, and provide support for its suitability in references [21-23]. Effectively, this is a modified Poisson model with a log link of the form:

log(y­_i_) = β_0_ + β_1_x_1_ +…+ β_k_x_k_.

The outcome is assumed to be Poisson distributed, but with a very low probability of values >1, such that no values of 2 and above are observed. This has been shown to provide equivalent coefficients to a Cox proportional hazards with equal follow-up times for all observations. The exponentiated coefficients provide a measure of the relative risk, which in the case of binary, cross-sectional outcome data represent the ratio of prevalences between exposure groups. The model-estimated standard errors are, however, too wide, unless robust or scaled errors are used.
